# Efficacy and safety profile of 2^nd^ line Anti TB drugs in DRTB patients in a tertiary care hospital of South Punjab

**DOI:** 10.12669/pjms.40.11.8605

**Published:** 2024-12

**Authors:** Hafiz Muhammad Rizwan, Moazzam Ali Atif, Mazhar Hussain, Rimsha Nazeer

**Affiliations:** 1 Hafiz Muhammad Rizwan, FCPS. Department of Pulmonology, Sheikh Zayed Medical College/Hospital, Rahim Yar Khan, Pakistan; 2 Moazzam Ali Atif, FCPS. Department of Pharmacology, Sheikh Zayed Medical College/Hospital, Rahim Yar Khan, Pakistan; 3 Mazhar Hussain, M.Phil. Department of Pharmacology, Sheikh Zayed Medical College/Hospital, Rahim Yar Khan, Pakistan; 4 Rimsha Nazeer, FCPS. Department of Pulmonology, Sheikh Zayed Medical College/Hospital, Rahim Yar Khan, Pakistan

**Keywords:** Tuberculosis, Multidrug-Resistant Tuberculosis, Weight Alterations, Sputum Smear Grading, Adverse Drug Reaction

## Abstract

**Objective:**

The objective of the study was to assess the efficacy and safety profile of 2nd line Anti TB drugs in DR TB patients in a tertiary care hospital of South Punjab.

**Method:**

This retrospective cohort study included patients who received therapy for multidrug-resistant tuberculosis (MDR-TB) between March 2018 and June 2021 at the Pulmonology Department of Sheikh Zayed Hospital, Rahim Yar Khan. Sociodemographic data, TB treatment history, treatment schemes, safety profiles, weight measures, and sputum smear results were obtained from medical records. Outcome variables of interest included body weight, sputum smear grading for treatment efficacy, and ADRs for safety assessment.

**Results:**

The study showed a progressive decline in positive sputum smear results over the 24-month treatment for MDR-TB, with an increase in negative smear results from 33 (9.50%) at baseline to 97 (28.0%) by Month 24. Furthermore, the mean body weight significantly increased every three months throughout the trial. Beginning at 43.83 kg at baseline, the mean weight increased to 50.02 kg by the end of 24 months. ADRs were mostly mild to moderate, including depression (18.4%), arthralgia (17.1%), anxiety (16.5%), and Qtc prolongation (11.0%). The treatment adhered to international guidelines, using a tailored combination of second-line drugs and adjusting regimens based on drug resistance patterns from sputum culture and DST.

**Conclusion:**

The study findings suggest the efficacy of second-line anti-TB drug regimens in achieving sputum smear conversion and positive treatment outcomes in patients with MDR-TB. However, the occurrence of ADRs highlights the need for careful monitoring and management during treatment.

## INTRODUCTION

The World Health Organisation (WHO) has identified tuberculosis (TB) as a major worldwide health hazard, claiming thousands of lives each year. TB claimed the lives of 1.6 million people in 2021 alone. TB is the world’s second most prevalent infectious cause of death, surpassing even HIV and AIDS, and is the 13th leading cause of death.^1^ Drug-resistant tuberculosis (DR-TB) poses a serious threat to public health in Pakistan, weakening efforts to control and prevent the illness.^2^,^3^ Rifampicin-resistant tuberculosis (RRTB), multidrug-resistant tuberculosis (MDR-TB), and extensively drug-resistant tuberculosis (XDR-TB) are all examples of this.

Pakistan ranks fifth in the incidence of drug-susceptible tuberculosis (DS-TB) and fourth in the incidence of drug-resistant tuberculosis (DR-TB).^4^ Over the last decade, the primary driver of DR-TB has been an increase in resistant strains of Mycobacterium tuberculosis, particularly against isoniazid and rifampicin.^5^ While first-line anti-TB medications (FLDs) such as isoniazid, rifampicin, ethambutol, and pyrazinamide effectively cure DS-TB, treating DR-TB necessitates a combination of SLDs. Fluoroquinolones (levofloxacin and moxifloxacin), aminoglycosides (amikacin, kanamycin, and capreomycin), and other key SLDs such as ethionamide, prothionamide, cycloserine, linezolid, and clofazimine are among these SLDs. Along with FLDs that remain effective against the TB bacterium, additional medicines such as bedaquiline and delamanid may be used in the therapy regimen.^6^

The treatment of multidrug-resistant tuberculosis entails the long-term administration of multiple drugs, which are becoming increasingly expensive and difficult to manage. Adherence to treatment guidelines and correct microbiological diagnosis are critical determinants in DR-TB patient outcomes. Anti-TB SLDs not only have lower efficacy than FLDs but they are also associated with more frequent and potentially fatal adverse drug reactions (ADRs). ADRs can vary in kind and frequency among patients receiving anti-TB medications, potentially leading to morbidity and fatality if not discovered early in the treatment process.

Tuberculosis infectiousness is proportional to the concentration of bacilli in the sputum, as indicated by sputum smear grading and the length of infectiousness. Individuals with higher sputum acid-fast bacilli (AFB) grades are more likely to spread the disease and have a higher chance of acquiring active TB among their contacts than individuals with lower grading, according to research.^7^ Furthermore, higher sputum smear grades are related to poor treatment outcomes and increased mortality rates in tuberculosis patients.^8^ As a result, those with a high sputum AFB grade are potential sources of continued TB transmission and contribute to the spread of drug-resistant TB in communities. This significantly complicates TB prevention and control measures.

Moreover, body weight changes have emerged as a potential prognostic indicator for tuberculosis treatment, particularly in drug-sensitive TB.^9^ Routine weighing of patients during follow-up visits to evaluate treatment response has been implemented in various countries with standardized treatment protocols. Consequently, body weight measurement holds promise as a valuable tool for predicting TB treatment outcomes. However, the potential association between body weight variation and treatment outcomes in multidrug-resistant tuberculosis (MDR-TB) cases remains unexplored.

In the light of these knowledge gaps, our study aimed to evaluate the efficacy and safety profile of second-line anti-TB drug regimens in DR-TB patients treated at a tertiary care hospital in South Punjab.

## METHOD

A retrospective cohort analysis was conducted utilizing data from patients starting MDR-TB therapy between March 2018 and June 2021, in accordance with the Strengthening the Reporting of Observational Studies in Epidemiology (STROBE) criteria. Data were gathered from the Sheikh Zayed Hospital Rahim Yar Khan’s Pulmonology Department. Sociodemographic data, TB treatment history, treatment plan, safety profile, weight measurements, and sputum smear results were gathered and evaluated in the medical records.

Patients with pulmonary multidrug-resistant tuberculosis (MDR-TB) who were 18 years old or above were included in the study. Patients who dropped out of therapy or had treatment failure throughout the follow-up period were not included in the statistical analysis. Patients with mono, poly, extensively drug-resistant tuberculosis (XDR-TB) or extra-pulmonary MDR-TB were also barred from participating. Patients with unknown or unclear medication resistance patterns were also excluded from the study.

### Outcomes and variables of interest:

The primary outcome variables were body weight and sputum smear grading assessed for determining the treatment efficacy, while for safety, ADRs were evaluated using World Health Organization (WHO) standard criteria. A systematic proforma was used to record pharmacovigilance data on adverse effects. The date, time, duration, intensity, culprit substance, spontaneous resolution, and whether supportive or aggressive treatment was administered were all collected. Other variables of interest included in the analysis were age, gender, previous tuberculosis FLD and SLD treatment history, treatment strategy, hospital duration, etc.

### Treatment Procedures:

Three hundred thirty-one patients were put on a long treatment regimen (LTR), which comprised of treatment duration of 18-24 months, and 169 patients who met the criteria for a shorter treatment regimen (STR) were put on this short regimen of 9 to 11 months as shown in Table-I with a respective regimen. Sputum samples were evaluated for smear microscopy after Zielh-Neelson (ZN) staining and GeneXpert/Rif. Patients with positive sputum smear and Rifampicin resistance detected on GeneXpert were selected for DR-TB treatment initiation. After the results of sputum culture and Drug susceptibility testing (DST), the treatment regimen was tailored according to specific resistance patterns. Complete blood count (CBC), liver function test (LFTs), renal function test (RFTs), serum electrolytes (S/E), random blood sugar (RBS), and uric acid tests were performed on a monthly basis. Thyroid function tests (TFTs) & HIV screening were conducted at the start of treatment. Visual and hearing function tests were conducted when needed.

**Table-I:** Clinical Characteristics of Patients with Multidrug-Resistance Tuberculosis.

Variables				(n=681)
Age (years)				38.93±17.5
Age Group			<35 years	313(46.0)
			35 to 64 years	300(44.1)
			≥ 65 years	68(10.0)
Gender			Male	388(57.0)
			Female	292(42.9)
			Transgender	1(0.1)
Previous TB Treatment History	FLD	Not reported		264(38.8)
		HREZ		331(48.60)
		HREZS		85(12.48)
		N/A		1(0.1)
	SLD	No		633(93.0)
		Yes		48(7.0)
Registration group			New	235(34.5)
			Others previously treated	223(32.7)
			Previously treated (after failure)	105(15.4)
			Previously treated (After the loss to follow-up)	23(3.4)
			Previously treated (Relapse)	69(10.1)
			Transfer in	20(2.9)
			Unknown previous treatment history	6(.9)
Diagnostic culture/DST			Not reported	115(16.9)
			Contaminated	54(7.9)
			Negative	84(12.3)
			Not done	5(0.7)
			Positive	422(62.0)
			Unknown	1(0.1)
Diagnostic Xpert test			Not reported	3(0.4)
			Not applicable on the basis of DST and Culture Report	1(0.1)
			Not done	2(0.3)
			Other	1(0.1)
			Tb not resistant to R	18(2.6)
			TB resistant to R	656(96.3)
Type of DR-TB resistance now			Not reported	1(0.1)
			MDR	383(56.2)
			Mono	9(1.3)
			Poly	2(0.3)
			Pre-XDR	78(11.5)
			XDR	21(3.1)
			Xpert MTB/RIF	187(27.5)
Treatment strategy			LTR	331(48.6)
			LTR-1	52(7.6)
			LTR-2	59(8.6)
			LTR-2a	25(3.7)
			LTR-2b	24(3.5)
			LTR-3	18(2.6)
			LTR-4	3(0.4)
			STR	169(24.8)
Initial Treatment Regimen			None	357(52.4)
			(4-6)am,mfx,eto,cfz,e,h-inh,z,b6/5 mfx,cfz,e,z,b6	15(2.2)
			(2)Lzd-bdq-lfx-cfz-z-e-h/4bdq-lfx-cfz-z-e-h/3lfx-cfz-z-e	107(15.7)
			(6)lfx,lzd,cfz,cs,bdq, (6)z,b6/12,lfx,cfz,cs,z,b6	38(5.5)
			(6)bdq,mfx,eto,cfz,z,h,e,b6/5mfx,cfz,z,e,b6	84(12.3)
			6z,Lfx,Cs,Eto,Cfz,Lzd/12z,Lfx,Cs,Eto,Cfz	9(1.3)
			Others	71(10.4)
Current Treatment Regimen			(4-6)am,mfx,eto,cfz,e,h-inh,z,b6/5 mfx,cfz,e,z,b6	52(7.6)
			(9)Bdq,6am,Cs,Lzd,Cfz,Z,B6/9 Cs,Lzd,Cfz,Z,B6	18(2.6)
			(9)Bdq,6am,E,Cs,Lzd,Cfz,Z,B6/9bdq, Cs,E,Cfz,Z,B6	15(2.2)
			(2)Lzd-Bdq-Lfx-Cfz-Z-E-H/4bdq-Lfx-Cfz-Z-E-H/3lfx-Cfz-Z-E	70(10.2)
			(6)Lfx,lzd,cfz,cs,bdq, (6)z,b6/12,lfx,cfz,cs,z,b6	24(3.5)
			(6)Bdq,Mfx,Eto,Cfz,Z,H,E,B6/5mfx,Cfz,Z,E,B6	35(5.1)
			(8)am,Lfx,Cs,Eto,Z,B6 /12 Lfx,Cs,Eto,Z,B6	14(2.0)
			(8)am,Lfx,Eto,Cs,Pas,Z,B6/12 Lfx,Eto,Cs,Pas,Z,B6	17(2.4)
			(8)am,Lfx,Eto,Cs,Z,B6/12 Lfx,Eto,Cs,Z,B6	24(3.5)
			(8)am,Mfx,Cs,Eto,Bdq, (6)Z,B6/16 Cs,Eto,Mfx,Z,B6	14(2.0)
			(8)am,Mfx,Eto,Cs,Pas,Z,B6/16 Mfx,Eto,Cs,Pas,Z,B6	12(1.7)
			Others	386(56.6)

All patients were treated free of cost with monthly financial support and transport charges. Adverse drug reactions (ADRs) were identified and recorded during each follow-up visit. As per protocol, the MDR physician was required to closely monitor ADRs regularly on a monthly basis. They were either subjective (reported by the patients), objective (observed by the MDR physician), or some impairment in laboratory values. ADRs were managed as recommended by NTP criteria for programmatic management for drug resistance TB.^10^,^11^

### Data Analysis:

The acquired data was analyzed using SPSS version 22.0. Descriptive statistics were used for data presentation, with categorical variables reported as frequencies and percentages and continuous data as mean with standard deviation.

### Ethical Considerations:

To ensure the study was done ethically, the institutional review board (Ref# 616/IRB/SZMC/SZH; Date: 10-01-2023) granted ethical permission. Patient confidentiality was ensured by eliminating all identities and analyzing only anonymous data.

## RESULTS

### Baseline Characteristics:

For this study purpose, 681 patient files were available and included for review; baseline characteristics are reported in Table-I. Patients presented with MDR-TB at a mean age of 38.93 ± 17.5 years, and 57.0% were males. Around 34.5% were new patients, 32.7% were previously treated, and 15.4% were previously treated (after failure), while the remaining 17.4% included those previously treated (after loss to follow up), previously treated (relapse), transfer in, and those with unknown previous treatment history.

### Safety Profile:

From the 681 patient files, the safety data for 110 was obtained. Overall, 538 reports of ADRs were captured for 110 patients; four patients had at least one ADR, six had two ADRs, 14 reported having three ADRs, and 86 had more than three. Depression was the most common ADR, (18.4%) followed by arthralgia (17.1%), anxiety (16.5%), and Qtc prolongation (11.0%). The majority of ADRs were possibly or probably attributed to one or more anti-tuberculosis drugs. Furthermore, the cause and management of these DRs are presented in Table-II.

**Table-II:** Frequency and management of ADRs among DR-TB patients.

Adverse drug reaction	Suspected culprit/ supportive treatment	ADR n(%)	ADRs attributed to one or more anti-tuberculosis drugs			Action taken, management, and any adjustment in treatment regimen			

			Not related	Possible	Probable	Dose not changed	Dose reduced	Drug interrupted	Drug withdrawn
Acute renal injury	Am	2(0.4)	0	2	0	0	0	0	2
Allergic reaction	Cfz	1(0.2)	0	1	0	0	0	0	1
Anxiety	CBT, Cs, Es	89(16.5)	22	65	2	87	0	2	0
Arthralgia	Z, Diclo	92(17.1)	1	89	2	82	3	3	4
Auditory injury	Am	12(2.2)	0	9	3	1	9	1	1
Cough	-	1(0.2)	1	0	0	1	0	0	0
Depression	CBT, Cs, Es	99(18.4)	24	74	1	98	0	1	0
Dyspnea	Ventolin	1(0.2)	1	0	0	1	0	0	0
Electrolyte imbalance	Motilium, Risek, Syrup Lifcid	2(0.4)	2	0	0	2	0	0	0
Gastritis	Lfx	3(0.6)	0	3	0	0	3	0	0
GI Irritation	Risek, Syrup Lifcid Maxolon, Mfx, Motillium, Lfx, Eto	14(2.6)	2	12	0	12	1	1	0
Hemoptysis	Transamine, Amoxil, Syrup Hyderline	2(0.4)	2	0	0	2	0	0	0
Headache	Diclo, Vidaylin	1(0.2)	0	1	0	1	0	0	0
Hearing loss	Am	1(0.2)	0	1	0	1	0	0	0
Hepatitis	Z, Motilium, Risek, Syrup Lifcid	36(6.7)	2	34	0	33	0	0	3
Myelosuppression	Vidaylin, Theragran, Lzd	51(9.5)	16	35	0	48	0	0	3
Peripheral Neuropathy	Z, Theragran, Lzd, Vidaylin, Lfx	46(8.6)	12	34	0	44	0	1	1
Psychosis	Cs, Es	4(0.7)	0	3	1	1	0	2	1
Qtc prolongation	Bdq, Lzd	59(11.0)	2	57	0	49	0	1	9
Retinopathy	E, Lzd	4(0.7)	0	4	0	1	0	0	3
Skin discoloration	Z, Loratadine, Cfz, Pirriton, Cream Betaderm N	17(3.2)	0	17	0	6	3	1	7
Other	-	1(0.2)	0	1	0	0	0	0	1

Lfx-Levofloxacin; Cs- Cycloserine; Es-Escitalopram, Z-Zurig, Am-Amikacin, Mfx-Moxifloxacin; Eto-Ethionamide, Lzd-Linezolid, Bdq-Bedaquiline.

It was found that more than 50% of ADRs were recovering or recovered. Severity assessment was done by Hartwig and Siegel scale; the majority (50.18%) of ADRs were mild, 38.66% were moderate, and 11.15% of ADRs were severe (Table-III).

**Table-III:** Outcomes and Severity Assessment of adverse drug reactions.

	Outcome				Severity			Seriousness			

Adverse drug reaction	Not reported	Not recovered/ not resolved	Recovered/ resolved	Recovering/ resolving	Mild	Moderate	Severe	Not reported	Hospitalization (caused or prolonged)	Not serious	Permanent disability
Acute renal injury	1	0	1	0	2	0	0	0	0	2	0
Allergic reaction	0	0	0	1	0	1	0	0	0	1	0
Anxiety	8	3	20	58	57	23	9	0	0	89	0
Arthralgia	14	13	25	40	43	32	17	0	0	92	0
Auditory injury	3	9	0	0	5	5	2	0	0	2	10
Cough	0	0	0	1	1	0	0	0	0	1	0
Depression	11	7	19	62	32	61	6	1	0	98	0
Dyspnea	1	0	0	0	1	0	0	0	0	1	0
Electrolyte imbalance	1	0	0	1	1	0	1	0	0	2	0
Gastritis	0	0	1	2	0	2	1	0	0	3	0
Gi irritation	2	1	9	2	9	5	0	0	0	14	0
Hemoptysis	0	1	1	0	2	0	0	0	0	2	0
Headache	0	0	0	1	0	1	0	0	0	1	0
Hearing loss	0	1	0	0	1	0	0	0	0	0	1
Hepatitis	5	4	17	10	33	3	0	0	0	36	0
Myelosuppression	6	16	14	15	27	20	4	0	0	51	0
Peripheral neuropathy	4	7	7	28	28	11	7	0	0	46	0
Psychosis	0	2	1	1	2	0	2	0	2	2	0
Qtc prolongation	9	7	33	10	20	29	10	0	0	59	0
Retinopathy	0	2	0	2	0	3	1	0	0	4	0
Skin discoloration	2	9	3	3	5	12	0	0	0	17	0
Other	0	0	1	0	1	0	0	0	0	1	0

### Body Weight Variation:

**Fig.1:**
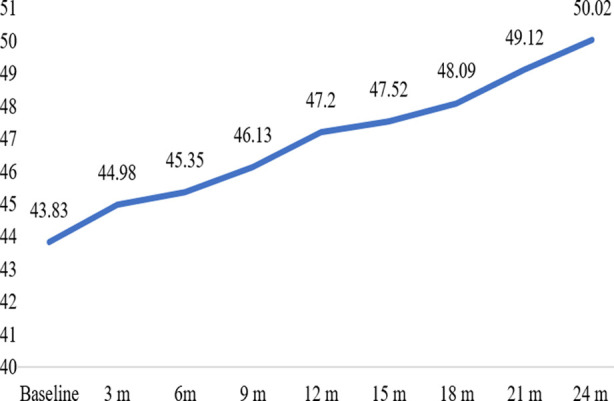
Increase in mean body weight of MDR-TB patients at different time intervals.

During the medication therapy, the mean body weight significantly increased every three months throughout the trial. Beginning at 43.83 kg at baseline, the mean weight increased to 50.02 kg by the end of 24 months (Fig.1).

### Sputum Smear Grading:

The distribution of sputum smear grading among MDR-TB patients from baseline to month 24 is presented in Table-IV. At baseline, the cured patients had a smear grading of 1+, while those who died had a smear grading of 2+. After 21-24 months, most of the smear grading reports were negative.

**Table-IV:** Distribution of sputum smear grading among MDR-TB patients at different time intervals.

		Treatment outcome						

		Complete	Cured	Died	Failed	Lost to follow up	Still under treatment	Treatment not evaluated
Baseline	Scanty	-	44(12.70)	19(11.90)	2(20.0)	2(5.90)	15(14.0)	5(22.70)
	+	-	128(37.0)	44(27.70)	2(20.0)	16(47.10)	37(34.60)	4(18.20)
	++	1(33.30)	92(26.60)	48(30.20)	1(10.0)	7(20.60)	28(26.20)	5(22.70)
	+++	-	49(14.20)	34(21.40)	4(40.0)	6(17.60)	23(21.50)	5(22.70)
	Negative	2(66.70)	33(9.50)	13(8.20)	1(10.0)	3(8.80)	4(3.70)	3(13.60)
	Not done	-	-	1(0.60)	-	-	-	-
Month 3	Scanty	-	1(0.30)	4(2.50)	-	1(2.90)	-	-
	Pending reports*	1(33.30)	2(0.60)	79(49.70)	-	22(64.70)	27(25.20)	14(63.60)
	+	-	1(0.30)	2(1.30)	-	-	-	-
	++	-	1(0.30)	-	-	-	-	-
	Negative	-	331(95.70)	70(44.00)	10(100.0)	7(20.60)	79(73.80)	8(36.40)
	Not done	2(66.70)	10(2.90)	4(2.50)	-	4(11.80)	1(0.90)	-
Month 6	Scanty	-	-	1(0.60)	-	-	-	-
	Pending reports*	1(33.30)	-	109(68.60)	-	26(76.50)	56(52.30)	17(77.30)
	+	-	1(0.30)	1(0.60)	-	-	-	-
	Negative	-	335(96.80)	45(28.30)	10(100.0)	5(14.70)	50(46.70)	5(22.70)
	Not done	2(66.70)	10(2.90)	3(1.90)	-	3(8.80)	1(0.90)	-
Month 9	Pending reports*	1(33.30)	2(0.60)	128(80.50)	1(10.0)	31(91.20)	79(73.80)	21(95.50)
	++	-	-	1(0.60)	1(10.0)	-	-	-
	+++	-	1(0.30)	-	-	-	-	-
	Negative	-	335(96.80)	28(17.60)	8(80.0)	2(5.90)	28(26.20)	1(4.50)
	Not done	2(66.70)	8(2.30)	2(1.30)	-	1(2.90)	-	-
Month 12	Pending reports*	1(33.30)	82(23.70)	143(89.90)	4(40.0)	33(97.10)	88(82.20)	22(100.0)
	+	-	-	1(0.60)	-	-	-	-
	+++	-	2(0.60)	-	-	-	-	-
	Negative	-	257(74.30)	14(8.80)	5(50.00)	1(2.90)	19(17.80)	-
	Not done	2(66.70)	5(1.40)	1(0.60)	1(10.0)	-	-	-
Month 15	Scanty	-	-	-	1(10.0)	-	-	-
	Pending reports*	1(33.30)	83(24.0)	150(94.30)	6(60.0)	34(100.0)	93(86.90)	21(95.50)
	Negative	-	258(74.60)	9(5.70)	3(30.0)	-	14(13.10)	1(4.50)
	Not done	2(66.70)	5(1.40)	-	-	-	-	-
Month 18	Pending reports*	1(33.30)	83(24.0)	154(96.90)	7(70.0)	34(100.0)	105(98.10)	21(95.50)
	+	-	1(0.30)	-	-	-	-	-
	++	-	-	1(0.60)	-	-	-	-
	Negative	-	257(74.30)	4(2.50)	3(30.0)	-	2(1.90)	1(4.50)
	Not done	2(66.70)	5(1.40)	-	-	-	-	-
Month 21	Scanty	-	-	1(0.60)	-	-	-	-
	Pending reports*	2(66.70)	190(54.90)	155(97.50)	9(90.0)	34(100.0)	107(100.0)	22(100.0)
	++	-	-	1(0.60)	-	-	-	-
	Negative	-	152(43.90)	2(1.30)	1(10.0)	-	-	-
	Not done	1(33.30)	4(1.20)	-	-	-	-	-
Month 24	Pending reports*	2(66.70)	247(71.40)	158(99.40)	10(100.0)	34(100.0)	107(100.0)	22(100.0)
	Negative	-	97(28.0)	1(0.60)	-	-	-	-
	Not done	1(33.30)	2(0.60)	-	-	-	-	-

* Pending reports due to lab errors, sample contamination, sent again to lab and results awaited, results of culture received from the lab after 1.5 months and DST received after 03 months.

## DISCUSSION

The current study focuses on the efficacy and safety aspects of the treatment among patients with multidrug-resistant tuberculosis (MDR-TB). The efficacy of the treatment was assessed through the analysis of weight variation and sputum smear grading at different time intervals. In terms of safety, this study examines the incidence of adverse drug reactions (ADRs), their severity, causes, and management.

In this study, 110 patients reported a total of 538 ADRs; four patients reported at least one ADR, six reported two ADRs, 14 reported three ADRs, and 86 reported more than three ADRs. One or more anti-tuberculosis medications were responsible for most of the reported ADRs. (Notably, around fifty percent of the ADRs were either recovering or were already recovering. Just a small portion of the ADRs were categorized as severe, with the majority of cases falling into the mild to moderate severity range. The most often reported ADRs were anxiety, arthralgia, and depression. The drugs associated with the highest ADRs were Cycloserine, Escitalopram, Zurig, Diclofenac, Bedaquiline, and Linezolid. Contrary to our findings, literature indicates that the most commonly affected systems are the digestive system and skin.^12^ Gastrointestinal symptoms, including nausea and vomiting, typically occurred earlier than skin manifestations during anti-tuberculosis therapy (ATT) and were sometimes associated with liver and biliary system disorders. These gastrointestinal reactions are likely related to rifampicin serum peaks, with symptoms generally subsiding as rifampicin blood concentrations decrease due to auto-metabolism.

One interesting finding from the Laghari et al. cohort was that 24.8% of patients had arthralgia without arthritis.^13^ This finding is consistent with the occurrence of arthralgia described in adult TB patients in India^14^ and the current study. Arthralgia, or joint pain, was also the most often seen adverse event in a different trial with 143 patients, accounting for 35.8% of the reported incidents. Other major adverse events that were described were gastrointestinal problems, ototoxicity, cutaneous responses, and dizziness.^15^ According to a study by Dela et al., 22.7% of ADRs were classified as severe, whereas 77.3% were mild to moderate in severity. Psychological ADRs, hearing loss, hepatotoxicity, and nausea/vomiting were examples of severe ADRs.^16^

In addition, our study discovered a significant association between the use of bedaquiline and linezolid and QTc prolongation (11%), raising questions about the safety of the heart during the treatment of drug-resistant tuberculosis (DR-TB). About 11% of the reported adverse drug reactions (ADRs) were associated with QTc prolongation. These results emphasize the need for careful cardiac function and QTc interval monitoring when bedaquiline and linezolid are prescribed to DR-TB patients. Similar research was done in South Africa by Isralls et al., who focused on a group of DR-TB patients who were receiving bedaquiline, clofazimine, and levofloxacin^17^ in a routine treatment environment.

They found that the QTc interval, a gauge of heart electrical activity, had slightly increased. The first four to six weeks of treatment saw the greatest QTcF interval lengthening, and week 15^16^ saw the greatest likelihood of the combination outcome. These findings imply that lengthening of the QTc interval may occur when these drugs are used to treat DR-TB. Therefore, it is essential to closely monitor heart function during the course of therapy. In a programmatic environment, evaluating patients with multidrug-resistant tuberculosis (MDR-TB) entails routine assessments of weight, sputum, and culture during the course of treatment. As well as monitoring bacteriological improvement during tuberculosis (TB) treatment, sputum smear and culture examination play a critical role in determining the bacteriological load and infectious status of patients. To designate patients as non-infectious, sputum culture conversion must be achieved within the first three months of treatment.^18^ Sputum culture conversion rates have been reported in prior international research to range from 74% to 92%.^19^,^20^

According to the 1+, 2+, or 3+ smear grading used in our investigation, the majority of patients exhibited a moderate to high bacterial burden at baseline. A lesser percentage of the samples produced insufficient data, which showed a very low bacterial load. The number of patients with positive smear results gradually decreased as therapy progressed, whereas the number of patients with negative smear results gradually rose. The majority of patients displayed negative smear findings by the conclusion of the 24-months therapy period, demonstrating an effective treatment response. Drug-resistant tuberculosis (DR-TB) patients with bacteriological confirmation had an overall sputum smear positivity rate of 83.65% in an Ethiopian research. This suggests that around 85% of lung DR-TB patients in the study area are able to transmit drug-resistant Mycobacterium tuberculosis strains to other people.

Additionally, we discovered in our study that 16% of the pulmonary DR-TB patients with bacteriological confirmation had a sputum smear grade of 2+ and more than one-third (34.42%) had a grade of 3+. In a research conducted in Mali, over two-thirds of MDR-TB patients were found to have a 3+ bacillary load.^21^ This prevalence was lower than that study’s findings. The study in Mali concentrated on MDR-TB patients who were hospitalized, whereas our study included all patients with pulmonary DR-TB who had been bacteriologically proven. This discrepancy may be due to differences in the study populations.

Our study’s percentage of patients with a 3+ sputum smear grade is consistent with research from India, where 39% of TB patients with positive smear tests had a 3+ smear grade.^22^ The likelihood of transmission rises with increasing smear grading, and it is widely known that patients with higher smear grades are more contagious.^23^ Due to a high Mycobacterium tuberculosis burden and prolonged contacts, high smear grading is frequently linked to treatment delay, which in turn causes broad transmission within communities.^24^

Hippocrates, who lived 2400 years ago, first used the term “phthisis” to describe tuberculosis, a classic wasting disease. This historical correlation emphasizes the value of weight as a proxy indicator of TB outcomes and patient development across time.^25^ Interestingly, although being so straightforward, the importance of weight as a predictor of treatment results has not been thoroughly researched. The mean body weight in the current study increased noticeably every three months till the end of the trial. According to this finding, weight may be a clinically useful predictor of TB treatment success. The therapeutic significance of weight in predicting treatment outcomes has also been investigated in other studies; some have recommended a cutoff of 5% weight increase to predict good TB treatment outcomes.^26^

Despite the fact that their research was limited to baseline and 12-month follow-up data, weight gain was found to be a poor predictor of treatment success. In a smaller sample of 30 participants, Schwenk et al. found a 10% increase in weight between the baseline and the sixth month of follow-up, primarily due to fat mass rather than protein mass.^26^

### Limitations:

There are some shortcomings to this study as well. The study employed a retrospective cohort design, which may be subject to inherent limitations such as incomplete or missing data, potential bias in data collection, and limited control over confounding factors. The reliance on medical records for data collection may introduce inconsistencies or errors in the recorded information. Further, the exclusion of patients with extensively drug-resistant tuberculosis (XDR-TB), extra-pulmonary MDR-TB, and those with unknown or undefined drug resistance patterns. This exclusion may lead to a potential selection bias and limit the generalizability of the findings to a broader DR-TB population. The accuracy and completeness of the recorded data in the medical records varied.

## CONCLUSION

In conclusion, the study observed that a substantial proportion of the enrolled patients had a moderate to high bacterial load at the start of treatment, as indicated by smear grading. As the treatment progressed, there was a gradual decline in the number of patients with positive smear results, indicating a positive treatment response. During the follow-up period, significant changes in body weight were observed among the patients. The study also reported the occurrence of ADRs among the patients. The majority of these ADRs were categorized as mild to moderate in severity. Various approaches, including pharmacological, non-pharmacological, and psychological interventions, were employed to manage these ADRs.

## References

[1] World Health Organization (WHO) Tuberculosis.

[2] Zafar N, Khaliq R, Memon M (2023). Rise of tuberculosis globally after a decline. J Pak Med Assoc.

[3] Jabbar A, Khan TA, Rehman H, Khan AS, Ahmad S, Khan SN (2021). Burden of Drug Resistant Tuberculosis in newly diagnosed Tuber-culosis patients of Khyber Pakhtunkhwa, Pakistan. J Pak Med Assoc.

[4] World Health Organization (WHO) (2021) WHO Global Lists of High Burden Countries for Tuberculosis (TB), TB/HIV and Multidrug/Rifampicin-Resistant TB (MDR/RR-TB), 2021–2025:Background Document.

[5] Alamgir M, Sajjad M, Baig MS, Noori MY (2021). Mutational Frequencies in Mycobacterial rpoB gene using GeneXpert/MTB Rif Assay in Rifampicin Resistant patients at a tertiary care setting in Urban Sindh, Pakistan:Analysis from a Five-Year Period. Pak J Med Sci.

[6] Falzon D, Schünemann HJ, Harausz E, González-Angulo L, Lienhardt C, Jaramillo E (2017). World Health Organization treatment guidelines for drug-resistant tuberculosis, 2016 update. Eur Respir J.

[7] Lohmann EM, Koster BF, Le Cessie S, Kamst-van Agterveld MP, Van Soolingen D, Arend SM (2012). Grading of a positive sputum smear and the risk of Mycobacterium tuberculosis transmission. Int J Tuberc Lung Dis.

[8] Brahmapurkar KP, Brahmapurkar VK, Zodpey SP (2017). Sputum smear grading and treatment outcome among directly observed treatment-short course patients of tuberculosis unit, Jagdalpur, Bastar. J Family Med Prim Care.

[9] Krapp F, Veliz JC, Cornejo E, Gotuzzo E, Seas C (2008). Bodyweight gain to predict treatment outcome in patients with pulmonary tuberculosis in Peru. Int J Tuberc Lung Dis.

[10] National Tuberculosis Control Program. Protocol for Treating MDR-TB/RR-TB with Shorter Treatment Regimen (STR) (2017). December.

[11] World Health Organization (2018). WHO treatment guidelines for multidrug- and rifampicin-resistant tuberculosis, 2018 update.

[12] Sant´Anna FM, Araújo-Pereira M, Schmaltz CA, Arriaga MB, de Oliveira RV, Andrade BB (2022). Adverse drug reactions related to treatment of drug-susceptible tuberculosis in Brazil:a prospective cohort study. Front Trop Dis.

[13] Laghari M, Talpur BA, Sulaiman SA, Khan AH, Bhatti Z (2020). Adverse drug reactions of anti-tuberculosis treatment among children with tuberculosis. Int J Mycobacteriol.

[14] Qureshi W, Hassan G, Kadri SM, Khan GQ, Samuel B, Arshad A (2007). Hyperuricemia and arthralgias during pyrazinamide therapy in patients with pulmonary tuberculosis. Lab Med.

[15] Hoa NB, Nhung NV, Khanh PH, Hai NV, Quyen BT (2015). Adverse events in the treatment of MDR-TB patients within and outside the NTP in Pham Ngoc Thach Hospital, Ho Chi Minh City, Vietnam. BMC Res Notes.

[16] Dela AI, Tank ND, Singh AP, Piparva KG (2017). Adverse drug reactions and treatment outcome analysis of DOTS-plus therapy of MDR-TB patients at district tuberculosis centre:A four year retrospective study. Lung India.

[17] Isralls S, Baisley K, Ngam E, Grant AD, Millard J (2021). QT interval prolongation in people treated with bedaquiline for drug-resistant tuberculosis under programmatic conditions:a retrospective cohort study. In Open Forum Infectious Diseases.

[18] Iqbal Z, Ali Khan M, Aziz A, Nasir SM (2022). Time for culture conversion and its associated factors in multidrug-resistant tuberculosis patients at a tertiary level hospital in Peshawar, Pakistan. Pak J Med Sci.

[19] Van DA, Salim MA, Das AP, Bastian I, Portaels F (2004). Results of a standardised regimen for multidrug resistant tuberculosis in Bangladesh. Int J Tuberc Lung Dis.

[20] Prasad R, Verma SK, Sahai S, Kumar S, Jain A (2006). Efficacy and safety of kanamycin, ethionamide, PAS, and cycloserine in multidrug resistant pulmonary tuberculosis patients. Indian J Chest Dis Allied Sci.

[21] Baya B, Achenbach CJ, Kone B, Toloba Y, Dabitao DK, Diarra B (2019). Clinical risk factors associated with multidrug-resistant tuberculosis (MDR-TB) in Mali. Int J Infect Dis.

[22] Patel J, Dave P, Satyanarayana S, Kumar AM, Shah A, Ananthakrishnan R (2013). Pretreatment sputum smear grade and smear positivity during follow-up of TB patients in Ahmedabad, India. Public Health Action.

[23] Huerga H, Sanchez-Padilla E, Melikyan N, Atshemyan H, Hayrapetyan A, Ulumyan A (2019). High prevalence of infection and low incidence of disease in child contacts of patients with drug-resistant tuberculosis:a prospective cohort study. Arch Dis Child.

[24] Htun YM, Khaing TM, Aung NM, Yin Y, Myint Z, Aung ST (2018). Delay in treatment initiation and treatment outcomes among adult patients with multidrug-resistant tuberculosis at Yangon Regional Tuberculosis Centre, Myanmar:a retrospective study. PLoS One.

[25] Schwenk A, Macallan DC (2000). Tuberculosis, malnutrition and wasting. Curr Opin Clin Nutr Metab Care.

[26] Schwenk A, Hodgson L, Wright A, Ward LC, Rayner CF, Grubnic S (2004). Nutrient partitioning during treatment of tuberculosis:gain in body fat mass but not in protein mass. Am J Clin Nutr.

